# Questions in Parliament

**Published:** 1918-07-13

**Authors:** 


### LONDON HOSPITAL NURSES.

### Questions in Parliament.

ON Thursday, July 4, in the House of Commons, Major CHAPPLE asked the Prime Minister whether his attention has been called to a system of farming-out of nurses in the London Hospital, under which nurses are taken from their training in the wards at the end of their second year, are paid 13s. per week, and sent out to nurse as trained nurses in private cases at £2 2s. per week, the hospital profiting by this means to the extent of over £6,000 per year before the war; and whether he intends to introduce legislation to protect nurses and patients from this system?

Mr. WALSH said: "I have been asked by my right hon. friend to reply to this question. The arrangements made by the London Hospital with their nurses are not a matter over which the Government has any control. There is no intention of introducing legislation on the subject."

Sir C. HENRY said: "Has the hon. gentleman satisfied himself with regard to the accuracy of the statements in the question?"

Captain CARR-GOMM asked if the statement referred to in the question was not rather of a controversial character, and the expression "farming-out," though perhaps picturesque, very unfair to an institution which has done much good for a great number of years.

Major CHAPPLE said: "Is the hon. gentleman not aware that the London Hospital is the only great hospital which takes its nurses from their training in the wards at the end of the second year and admittedly pays them only 13s. a week while it draws £2 2s. a week profit?"

The SPEAKER said: "This question should not have appeared on the paper at all. No Government Department has any control over the affairs of the London Hospital."

Major CHAPPLE: "On a point of order, am I not entitled to ask whether in a case of this kind such an abuse should be remedied by legislation? Am I not entitled to call attention to the fact that such farming-out exists, and to ask the Prime Minister whether he will introduce legislation to prevent it?"

The SPEAKER: "If my attention had been called to its preamble I should certainly have struck it out."

